# Grape Seed Proanthocyanidins Exert a Radioprotective Effect on the Testes and Intestines Through Antioxidant Effects and Inhibition of MAPK Signal Pathways

**DOI:** 10.3389/fmed.2021.836528

**Published:** 2022-01-24

**Authors:** Hui Shen, Jun Han, Chunlei Liu, Fei Cao, Yijuan Huang

**Affiliations:** ^1^Department of Central Laboratory, First Hospital of Jiaxing, Affiliated Hospital of Jiaxing University, Jiaxing, China; ^2^Department of Radiology, First Hospital of Jiaxing, Affiliated Hospital of Jiaxing University, Jiaxing, China; ^3^Department of Radiation Oncology, Chifeng Municipal Hospital, Chifeng Clinical Medical School of Inner Mongolia Medical University, Chifeng, China; ^4^Department of Radiotherapy, Changhai Hospital of Shanghai, First Affiliated Hospital of Naval Medical University, Shanghai, China

**Keywords:** proanthocyanidins, testis, intestine, radioprotection, MAPK

## Abstract

The testes and intestines are highly sensitive to ionizing radiation. Low-dose radiation can cause infertility and enteritis. However, there is a lack of safe and efficient radioprotective agents. This study aims to investigate the radioprotective effects of grape seed proanthocyanidins (GSPs) on testicular and intestinal damage induced by ionizing radiation. *In vitro*, GSPs reduced the apoptosis and proliferation inhibition of mouse testicular stromal cells TM3 and human small intestinal crypt epithelial cells HIEC induced by ionizing radiation, and alleviated DNA double-strand breaks. *In vivo*, GSPs ameliorated the pathological damage of the testes and intestines induced by ionizing radiation, and protected the endocrine function of the testes and the barrier function of the intestines. In addition, we preliminarily proved that the radioprotective effect of GSPs is related to its antioxidant effect and inhibition of MAPK signaling pathways. Our results indicate that GSPs are expected to be a safe and effective radioprotective drug.

## Introduction

With the widespread application of nuclear energy in industry, medical, military and other fields, the probability of exposure to ionizing radiation (IR) by radiologists, patients, and the public has gradually increased. Male spermatogenic cells and intestinal epithelial cells are extremely sensitive to ionizing radiation, especially spermatogonia (1) and intestinal crypt stem cells (2, 3). Twenty-five miiligray of ionizing radiation can cause the apoptosis of spermatogonia, which in turn affects spermatogenesis, and ultimately affects the number of sperm produced in the testis and the quality of sperm in the epididymis (4, 5). Ionizing radiation causes decrease of the daily sperm production, increase of the sperm deformity rate, decrease of the interstitial cells, and decrease of the overall weight of the testes (6, 7). Ionizing radiation causes block of the differentiation of intestinal stem cells into various functional cells, cause extensive necrosis of intestinal epithelial cells, damage the mechanical and immune barriers of intestinal epithelium, and cause severe gastrointestinal bleeding, diarrhea, bacteremia and electrolyte disorders, etc. (2, 3, 8). Therefore, the research on the radiation protection of the testes and intestines is of great significance.

At present, amifostine is the only radiation protection drug certified by the US Food and Drug Administration (FDA). It plays a role in radiation protection by scavenging free radicals. However, the obvious side effects limit the application of amifostine. At lower drug doses, amifostine can have toxic effects on spermatogonial stem cells and intestinal epithelial cells (9, 10). In order to develop safe and effective radiation protection agents, scholars at home and abroad have conducted a lot of research, including toll-like receptors ligands, estrogen preparations, cytokines, sulfhydryl-containing compounds, etc. However, in general, these substances have large side effects or poor effects (11–13).

When the body is exposed to ionizing radiation, a large number of free radicals can be produced, which in turn leads to oxidative damage to DNA, lipids and proteins in cells (14–16). Therefore, it is an effective way to protect against radiation damage by scavenging free radicals. Proanthocyanidins have strong antioxidant activity and free radical scavenging ability (17), which is expected to be a safe and effective radioprotectant. We have confirmed the protective effect of proanthocyanidins on radiation-induced lung injury in previous experiments (18). In addition, the toxic and side effects of proanthocyanidins are very small (17). The clinical trials that have been carried out include the effects of proanthocyanidins on blood sugar, blood lipids, cardiovascular disease, non-alcoholic fatty liver, dementia, vision, platelet function, etc. (19–29), confirming the safety of proanthocyanidins applied to the human body.

The MAPK signaling pathway is involved in the regulation of cell proliferation, differentiation and apoptosis (30). When cells are physically or chemically damaged, the MAPK signaling pathway will be activated, thereby inducing cell apoptosis. Inhibition of MAPK signaling pathway can protect cells against damage. In a study of ionizing radiation-induced skin damage, ionizing radiation activated the MAPK signaling pathway (31). In another study, phlorizin played a protective role against UV-induced skin damage by inhibiting the P38 and JNK signaling pathways (32). Proanthocyanidins also protect skin cells against damage induced by ultraviolet rays by down-regulating the MAPK signaling pathway (33, 34). In osteoarthritis (35, 36) and encephalitis (37, 38), proanthocyanidins also play a similar role. This study preliminarily explored the role of proanthocyanidins and MAPK signaling pathways in the testicular and intestinal injury induced by ionizing radiation.

## Materials and Methods

### Cells Experiment

1. TM3 (Mouse testicular stromal cells) and HIEC (Human small intestinal crypt epithelial cells) were purchased from American Type Culture Collection and cultured in DMEM medium containing 10% fetal calf serum. Cell incubator kept at 37°C with 5% CO2 and 95% humidity.
2. TM3 and HIEC cells were exposed to ^60^Co (Naval Medical University, Shanghai) with a dose of 5 and 8 Gy, respectively, at a dose rate of 1 Gy/min.
3. GSPs was purchased from Tianjin Peak Natural Products Research and Development co. LTD. (Tianjin, China). Different concentrations of GSPs-rich PBS (phosphate buffer saline) were given 1 h before ionizing irradiation (IR).
4. CCK-8 assay (Cell Counting Kit-8; Dojindo Laboratories, Kumamoto, Japan) was used to detect cell viability. TM3 and HIEC cells were cultured in 96-well plates for 24 h and pretreated with or without GSPs-rich PBS at 1 h before radiation and further cultured for 24 h after radiation.
5. Colony formation assay was used to detect cell proliferation as previous research (18). TM3 and HIEC cells were cultured in 6-well plates for 2 weeks, with number of 200 cells in non-IR group and 2,000 cells in IR and GSPs+IR group. The crystal violet needed for cloning dyeing was purchased from Bailingwei Technology Co., Ltd. Beijing, China.
6. A reactive oxygen species (ROS) detection kit (Beyotime Biotechnology, Shanghai, China) was used to detect intracellular ROS levels. TM3 and HIEC cells were processed according to the steps of the ROS detection kit, and then the relative content of ROS in the cells was detected with a microplate reader (TC1000-S 3-6550, Fengzhou Technology Co., Ltd., Dalian, China), with parameters of Ex488nm/Em525nm.
7. The DNA strand breaks were measured by using single-cell gel electrophoresis based on the method of Dubner et al. (39), quantitatively analyzed by CaspLab software.

### Mice Experiment

1. Mice (C57BL/6, 6-week-old, male) were purchased from Shanghai Ling Chang biological technology co., LTD (Shanghai China). All the experiments associated with mice were approved by the Laboratory Animal Center of Naval Medical University, Shanghai, China.
2. Local testes and intestines of all radiated mice were exposed to ^60^Co with a dose of 5 and 8 G y (or 27 Gy), respectively, at a dose rate of 1 Gy/min.
3. GSPs (30 mg/ml, 400 mg/kg weight) was delivered through gavage 1 h before IR and fed continuously by drinking (2 mg/ml) until 4 weeks after IR.
4. Hematoxylin-eosin (HE) and TdT-mediated dUTP Nick-End Labeling (TUNEL) staining was performed as previously described (40). Both of the testes were dissected 24 h after 5 Gy IR of the lower abdomen for HE and TUNEL staining. Jejunum was dissected 5 days after 8 Gy IR of the upper abdomen for HE and TUNEL staining.
5. The calculation methods of testicular sperm head count, testicular organ index, seminiferous tubule diameter, daily sperm production, epididymal sperm quality (sperm motility rate, sperm count and sperm deformity rate) and tubule differentiation index refer to previous literature (41–43). The time for spermatogonia to differentiate and develop into sperm is 29 days. The number of sperm heads in the testis, testicular organ index (weight of both testes/weight of mouse) and seminiferous tubule diameter was measured 29 days after IR, which reflected the damage of spermatogonia during IR. The time for spermatogenic stem cells to differentiate and develop into sperm is 56 days. The number of sperm heads in the testis, the quality of epididymal sperm (sperm motility rate, sperm count and sperm deformity rate) and tubule differentiation index (percentage of differentiated tubules) was measured 56 days after IR, which reflected the damage of spermatogenic stem cells during IR. Since sperm cell development spans 4.84 days, the final count of sperm heads divided by 4.84 is the daily sperm production.
6. ELISA kits (Westang Tech., Shanghai, China) were used to detect testosterone levels of testes and serum, collected 29 days after IR.
7. Malondialdehyde (MDA), Glutathione (GSH), Superoxide Dismutase (SOD) detection kits (Beyotime Biotechnology, Shanghai, China) were used to detect the content of MDA, GSH, and SOD in testis and intestines, collected 24 h after IR.
8. Proteins from testes and intestines for Western Blot were extracted by ProtecJETTM Mammalian Cell Lysis Reagent (Fermentas, Vilnius, Baltic, Lithuania). P38 (1:1,000), p-P38 (1:1,000), JNK (1:1,000), and p-JNK (1:1,000) antibodies were provided by Abcam Corporation. Tubulin (1:2,000) and the secondary antibody (1:5,000) were provided by Cell Signaling Technology Corporation.

### Statistical Analysis

Data were expressed as mean ± SD of three independent experiments and calculated using one-way ANOVA (Prism version 6.0 software). Student-Newman-Keuls *post-hoc* test was used to determine variance between groups. The difference between the groups was considered statistically significant when *P* < 0.05.

## Results

### Grape Seed Proanthocyanidins Have a Radioprotective Effect on TM3 Cells and HIEC Cells

**Figure 1:**
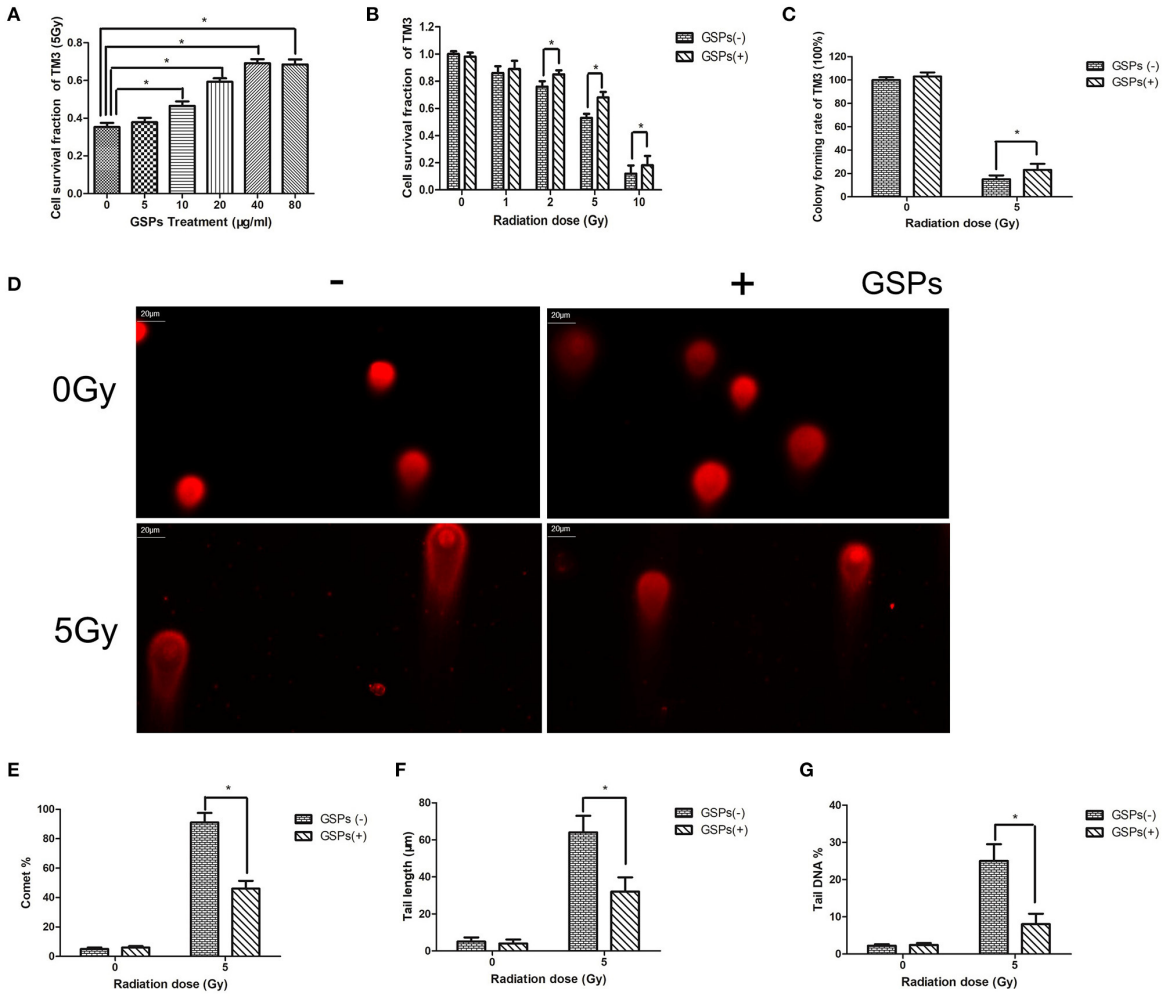
Grape seed proanthocyanidins have a radioprotective effect on mouse testicular stromal cells TM3. **(A)** When TM3 cells are exposed to 5 Gy ionizing radiation (IR), the radioprotective effect of grape seed proanthocyanidins (GSPs) is dose-dependent. **(B)** The viability of TM3 cells gradually decreases with the increase of the radiation dose, while GSPs increase the vitality of irradiated TM3 cells. **(C)** The clone formation assay shows that GSPs increase the proliferation ability of irradiated TM3 cells. **(D–G)** Effect of GSPs in TM3 cells on IR-induced DNA strand breaks assayed by comet assay. **(D)** Representative micrographs. **(E)** Comet%. **(F)** Tail length. **(G)** Tail DNA%. Data are presented as mean ± SD (*n* =6). *P < 0.05.

When TM3 cells were exposed to 5 Gy ionizing radiation, the radioprotective effect of grape seed proanthocyanidins was dose-dependent (Figure 1A). When the concentration of grape seed proanthocyanidins raise to 40 μg/ml, the radioprotective effect no longer increased with the increase of the drug concentration. The viability of TM3 cells gradually decreased with the increase of the radiation dose, while GSPs increased the vitality of irradiated TM3 cells (Figure 1B). The clone formation assay further verified the radioprotective effect of grape seed proanthocyanidins (Figure 1C). When TM3 cells were exposed to ionizing radiation, DNA double-strand broke, and grape seed proanthocyanidins can reduce DNA damage caused by ionizing radiation (Figures 1D–G).

**Figure 2:**
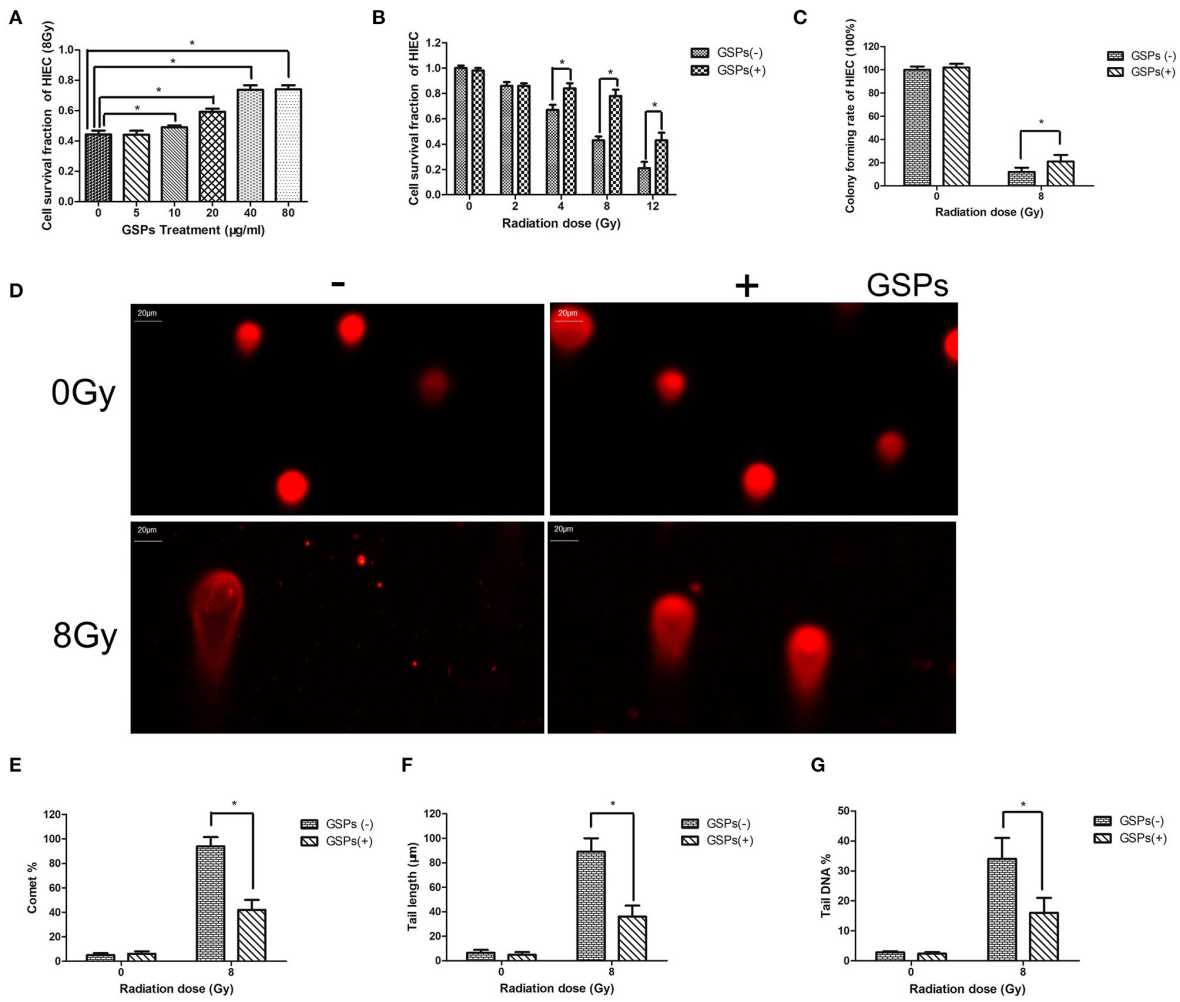
GSPs have a radioprotective effect on human small intestinal crypt epithelial cells HIEC. **(A)** When HIEC cells are exposed to 8 Gy IR, GSPs exert a dose-dependent radioprotective effect. **(B)** The viability of HIEC cells gradually decreases with the increase of the radiation dose, while GSPs increase the vitality of irradiated HIEC cells. **(C)** The clone formation assay shows that GSPs increase the proliferation ability of irradiated HIEC cells. **(D–G)** Effect of GSPs in HIEC cells on IR-induced DNA strand breaks assayed by comet assay. **(D)** Representative micrographs. **(E)** Comet%. **(F)** Tail length. **(G)** Tail DNA%. Data are presented as mean ± SD (*n* =6). *P < 0.05.

When HIEC cells were exposed to 8 Gy ionizing radiation, grape seed proanthocyanidins also exerted a dose-dependent radioprotective effect (Figure 2A). The viability of HIEC cells gradually decreased with the increase of the radiation dose, while GSPs increased the vitality of irradiated HIEC cells (Figure 2B). The clone formation assay (Figure 2C) and comet assay (Figures 2D–G) further verified the radioprotective effect of grape seed proanthocyanidins.

### Grape Seed Proanthocyanidins Have Radioprotective Effects on the Testes and Intestines

**Figure 3:**
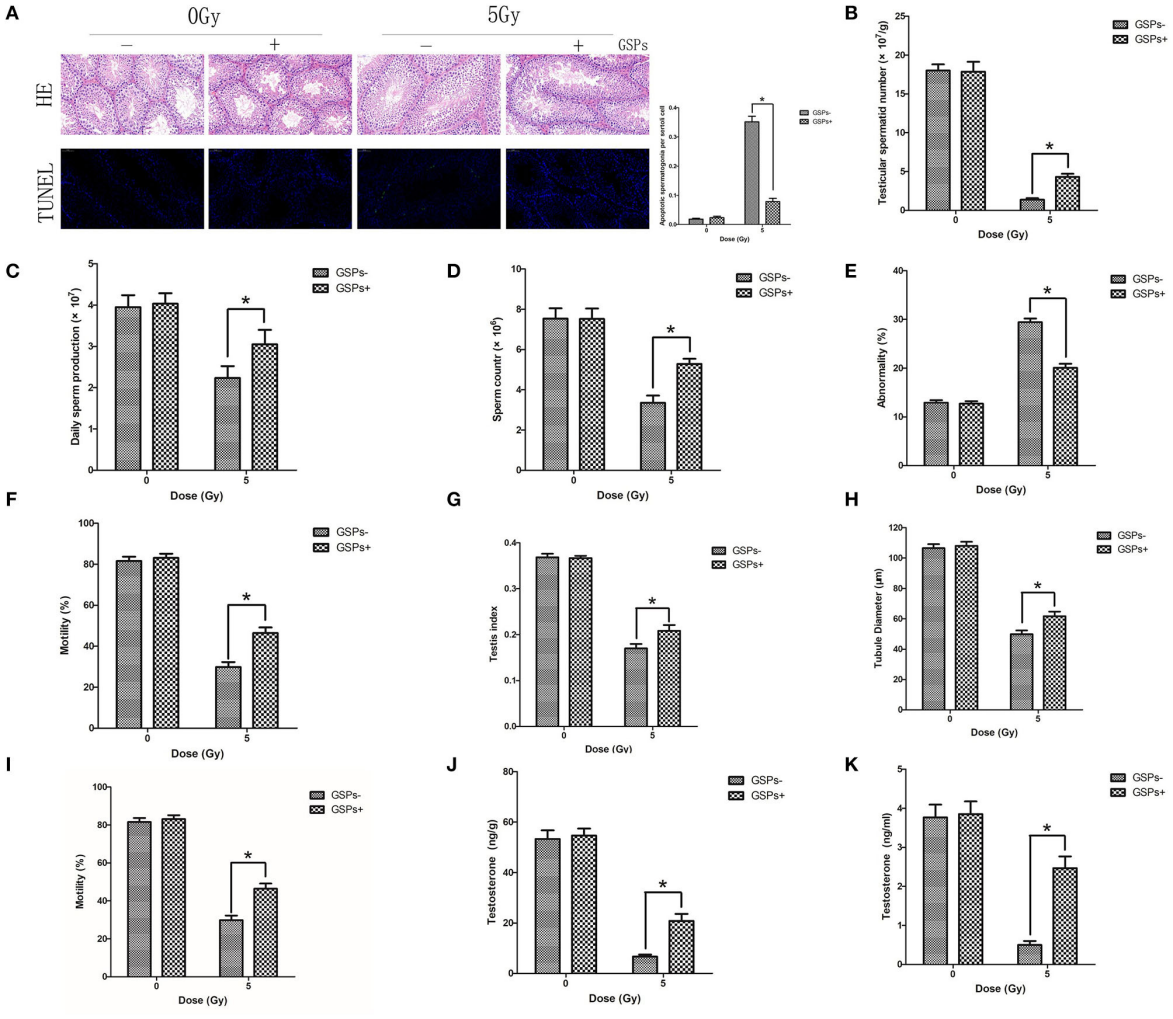
GSPs have radioprotective effects on the testes. **(A)** Hematoxylin-eosin (HE) and TdT-mediated dUTP Nick-End Labeling (TUNEL) staining of testes dissected 24 h after 5 Gy IR of the lower abdomen. HE staining shows no abnormality of sperm is found. TUNEL-positive cells only exist in spermatogonia. Quantitative analysis result shows that TUNEL-positive cells of the IR+GSPs group are significantly lower than those of the IR group. **(B)** The sperm head counts, 29 days post-IR, of the IR + GSPs group are significantly higher than those of the IR group. **(C)** The daily sperm production, 56 days post-IR, of the IR + GSPs group is significantly higher than that of the IR group. The sperm count **(D)** and mobility rate **(E)** of epididymis, 56 days post-IR, of the IR+GSPs group are significantly higher than those of the IR group, and the abnormality rate **(F)** is significantly lower than that of the IR group. **(G)** The testicular organ index (weight of both testes/weight of mouse), 29 days post-IR, of the IR+GSPs group is significantly higher than that of the IR group. **(H)** The diameter of the seminiferous tubules, 29 days post-IR, is significantly reduced, while GSPs alleviate this change. **(I)** The testicular tubule differentiation index (percentage of differentiated tubules), 56 days post-IR, of the IR + GSPs group is significantly higher than that of the IR group. ELISA assay shows that GSPs significantly alleviate the IR-induced decrease of the testosterone content in mouse testis **(J)** and serum **(K)**. Data are presented as mean ± SD (*n* = 6). *P < 0.05.

Both testes were dissected for HE and TUNEL staining (Figure 3A), 24 h after 5 Gy local irradiation of the lower abdomen. No abnormality of sperm was found in each group. TUNEL-positive cells only existed in spermatogonia, and no sperm was found to be positive in TUNEL staining. The positive rate of TUNEL staining of the IR + GSPs group was significantly lower than that of the IR group, indicating that GSPs can alleviate the apoptosis of spermatogenic cells induced by ionizing radiation. The sperm head counts in the testes were detected 29 days after ionizing radiation. The sperm head counts of the IR+GSPs group were significantly higher than those of the IR group (Figure 3B). The sperm head counts in the testes were detected 56 days after irradiation, and the daily sperm production was calculated. The daily sperm production of the IR+GSPs group was significantly higher than that of the IR group (Figure 3C). The quality of epididymal sperm was measured 56 days after irradiation, including sperm count, abnormality rate and mobility rate. The sperm count (Figure 3D) and mobility rate (Figure 3E) of the IR+GSPs group were significantly higher than those of the IR group, and the abnormality rate (Figure 3F) was significantly lower than that of the IR group. The weight of the mice and the weight of both testes were weighed, 29 days after irradiation, and the testicular organ index of the mice was measured. When the mice were irradiated with 5Gy, the testicular organ index of the IR + GSPs group was significantly higher than that of the IR group (Figure 3G). The diameter of the seminiferous tubules was measured with a micrometer, 29 days after irradiation, and the results showed that the diameter of the seminiferous tubules was significantly reduced, while GSPs alleviated this change (Figure 3H). The testicular tubule differentiation index was measured 56 days after irradiation. The testicular tubule differentiation index of the IR+GSPs group was significantly higher than that of the IR group (Figure 3I). Testosterone is a hormone secreted by testicular stromal cells, which plays an important role in promoting sperm production. ELISA assay shows that GSPs significantly alleviate the IR-induced decrease of the testosterone content in mouse testis (Figure 3J) and serum (Figure 3K).

**Figure 4:**
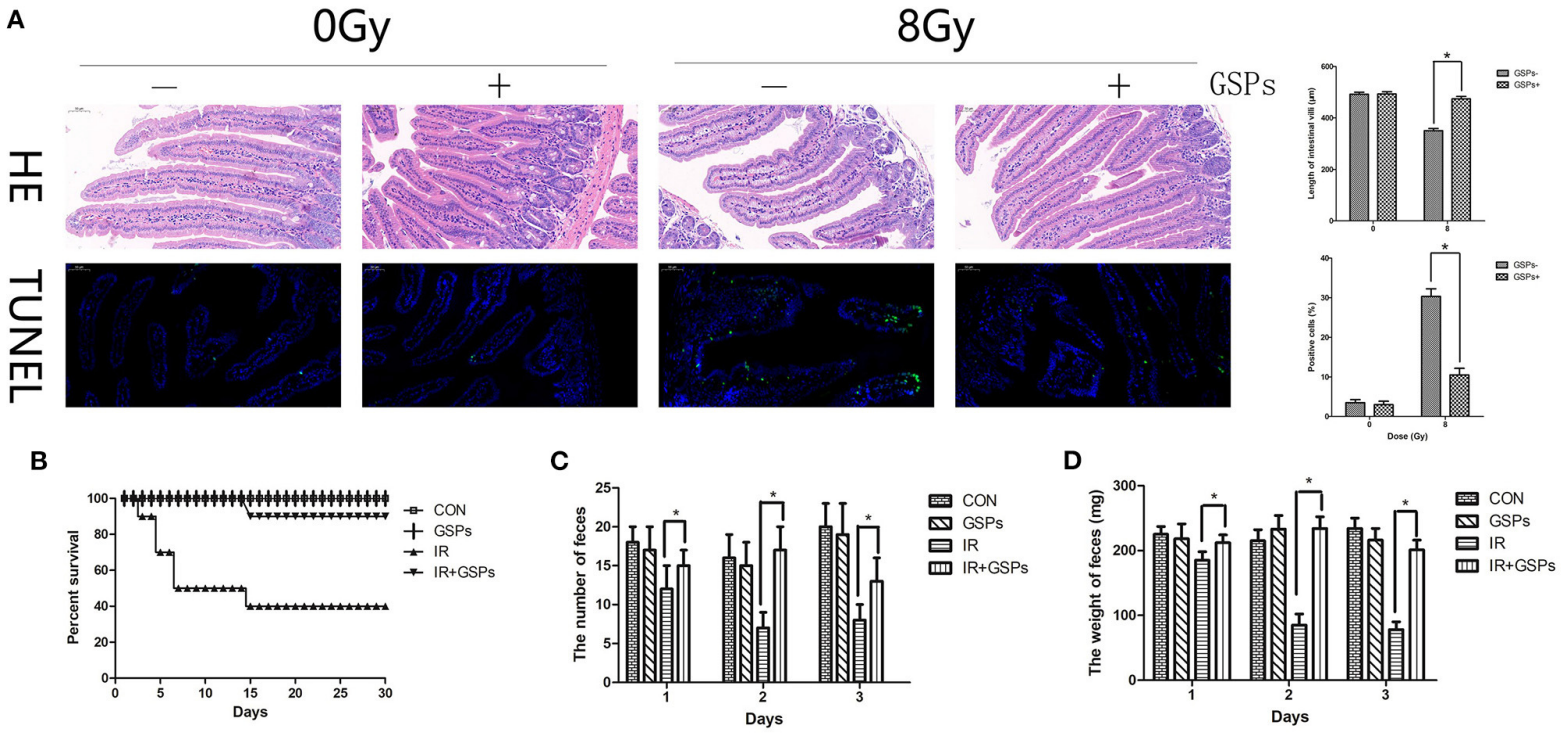
Grape seed proanthocyanidins have radioprotective effects on the intestines. **(A)** HE and TUNEL staining of the jejunum dissected 5 days after 8 Gy local abdominal IR. Quantitative analysis results show that the small intestinal villus is shortened induced by IR, with apoptosis of a large number of intestinal epithelial cells, while GSPs alleviate these injuries. **(B)** The 30-day survival rate of the IR group is 40% after 27 Gy abdominal local IR, while the 30-day survival rate of the IR+GSPs group is 90%. The quantity **(C)** and weight **(D)** of the feces in the first 3 days after 27 Gy IR are significantly reduced, while GSPs treatment alleviate these changes. Data are presented as mean ± SD (*n* = 10, Figure 5B; *n* = 6, Figures 5C,D). *P < 0.05.

The jejunum was dissected for HE and TUNEL staining 5 days after 8 Gy local abdominal irradiation (Figure 4A). The results showed that the small intestinal villi were shortened, with apoptosis of a large number of intestinal epithelial cells, while GSPs alleviated these injuries. The 30-day survival rate of mice was calculated after 27 Gy local abdominal irradiation (Figure 4B). The results showed that the 30-day survival rate of the IR group was 40%, while the 30-day survival rate of the IR+GSPs group was 90%, which was significantly higher than that of the IR group. In addition, we counted the changes in feces in the first 3 days of each group, and the results showed that the feces of mice changed significantly after ionizing radiation. The quantity (Figure 4C) and weight (Figure 4D) were significantly reduced, and the feces appeared to be non-particles and hydrated. However, the quantity and weight of feces in the IR+GSPs group did not decrease significantly, and there were no morphological changes such as hydration and deformation in the feces. Therefore, GSPs have a certain alleviating effect on intestinal dysfunction and feces formation difficulties caused by ionizing radiation.

### Grape Seed Proanthocyanidins Play a Radioprotective Effect Through Antioxidant Effects and Inhibition of MAPK Signaling Pathways

**Figure 5:**
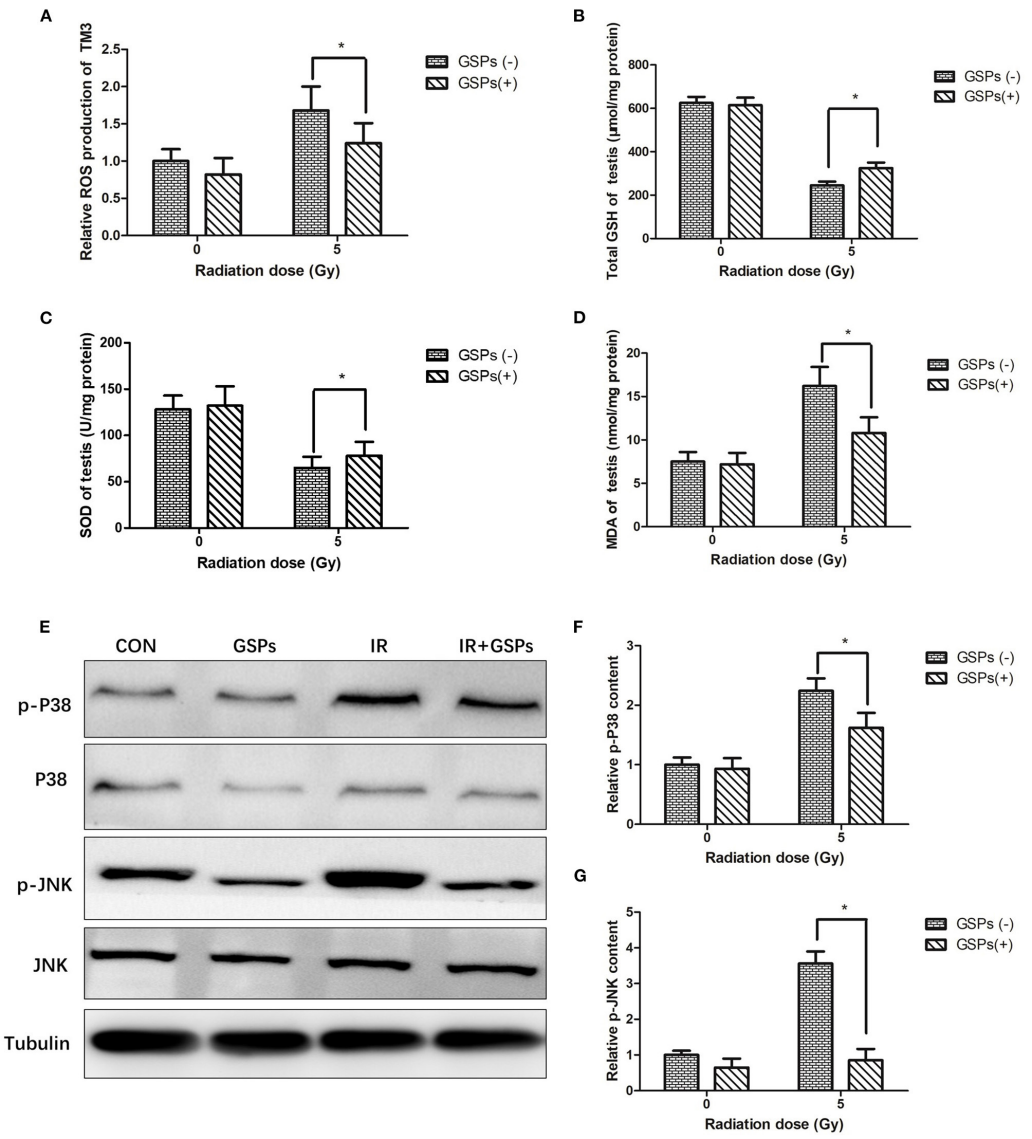
Grape seed proanthocyanidins play a radioprotective effect in testes through antioxidant effects and inhibition of MAPK signaling pathways. **(A)** The increase of intracellular ROS of TM3 cells induced by IR is significantly suppressed by GSPs. The content of total GSH **(B)** and SOD **(C)** in the testes decrease by IR, and the content of MDA **(D)** increases, while GSPs alleviate these changes. **(E)** The expression of p-P38 and p-JNK is significantly increased in irradiated testes, while GSPs lower the increase. **(F)** Quantitative analysis of relative p-P38 content. **(G)** Quantitative analysis of relative p-JNK content. Data are presented as mean ± SD (*n* = 3). *P < 0.05.

When TM3 cells were exposed to ionizing radiation, the level of intracellular ROS was significantly increased, and when TM3 cells were pretreated with GSPs and then subjected to ionizing radiation, the increase in intracellular ROS was significantly suppressed (Figure 5A). When mice were exposed to ionizing radiation, the content of GSH (Figure 5B) and SOD (Figure 5C) in the testes decreased, while the content of MDA (Figure 5D) increased. GSPs alleviated these changes and improved the oxidative stress state of the mouse testes. We dissected the mouse testes, and then performed Western Blot to detect the effects of IR and GSPs on the proteins related to the MAPK signaling pathway. The results showed that ionizing radiation activated the MAPK signaling pathway, and the expression of p-P38 and p-JNK was significantly increased, while GSPs decreased the expression of p-P38 and p-JNK (Figures 5E–G).

**Figure 6:**
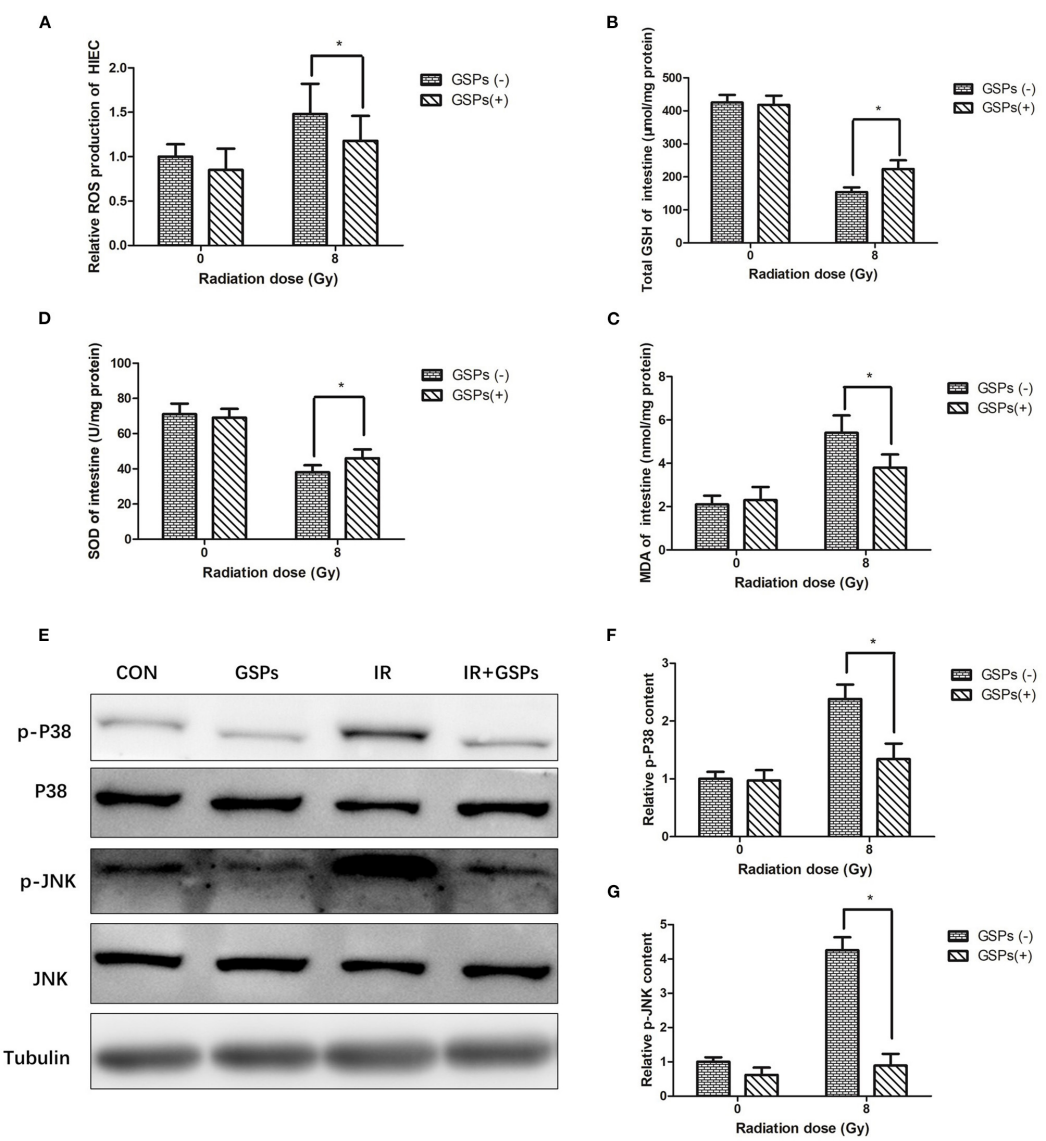
Grape seed proanthocyanidins play a radioprotective effect in intestines through antioxidant effects and inhibition of MAPK signaling pathways. **(A)** GSPs eliminate the increased ROS of HIEC cells induced by IR. The content of GSH **(B)** and SOD **(C)** in the intestines decrease by IR, and the content of MDA **(D)** increases, while GSPs alleviate these changes. **(E)** GSPs inhibit the IR-induced activation of the MAPK signaling pathway, manifested as down-regulation of the expression of p-P38 and p-JNK. **(F)** Quantitative analysis of relative p-P38 content. **(G)** Quantitative analysis of relative p-JNK content. Data are presented as mean ± SD (*n* = 3). *P < 0.05.

When HIEC cells were exposed to ionizing radiation, GSPs also eliminated the increased ROS in the cells caused by ionizing radiation (Figure 6A). When mice were treated with IR and GSPs, the changes in GSH (Figure 6B), SOD (Figure 6C), and MDA (Figure 6D) in the small intestine were similar to those in the testis. Similarly, the changes of the proteins associated with the MAPK signaling pathway in the intestinal tissues of mice were similar to those in the testis (Figures 6E–G).

## Discussion

The testes and intestines are highly sensitive to ionizing radiation, and low-dose radiation can cause infertility and enteritis, but there is still a lack of safe and efficient radioprotective agents. Because of its obvious antioxidant capacity, GSPs played a significant role in radioprotective effect on testicular and intestinal damage caused by ionizing radiation in this study.

Proanthocyanidins play a significant protective effect in oxidative stress-related diseases. For example, proanthocyanidins play a protective role in ultraviolet-induced keratinocyte damage (44), oxidative stress-induced renal tubular cell apoptosis (45), and ionizing radiation-induced liver damage (46) and lymphocyte damage (47). Our previous experiments also confirmed that proanthocyanidins have radioprotective effects on lung epithelial cells and AHH-1 cells (18, 48, 49). In this study, our results show that proanthocyanidins can reduce the damage to mouse testicular stromal cells TM3 and human small intestinal crypt epithelial cells HIEC caused by ionizing radiation, reduce cell apoptosis, promote cell proliferation, and reduce DNA double-strand breaks. This result is consistent with previous studies, further confirming the radioprotective effect of proanthocyanidins.

Spermatogenesis can be divided into three stages, including the proliferation of spermatogonia, the meiosis of spermatocytes, and the spermatogenesis. Sperm is relatively resistant to ionizing radiation, while spermatogonia are highly sensitive to ionizing radiation (50). Studies have shown that ionizing radiation with a dose as low as 25 mGy can cause the apoptosis of spermatogonia (4). In this study, ionizing radiation of 5 Gy cause apoptosis of the spermatogonia, but there was no obvious abnormality in the sperm. According to the proliferation and differentiation law of mouse germ cells, the time from spermatogenic stem cells to sperm is 56 days, and the time from spermatogonia to sperm is 29 days (51). Therefore, at these two time points, we used the following indicators to evaluate the proliferation and differentiation of germ cells after IR, including testicular sperm head count, daily sperm production, sperm motility rate, testicular organ index, seminiferous tubule diameter, testicular tubule differentiation index, testosterone content, etc. The results showed that ionizing radiation down-regulated these indicators, while GSPs greatly reduced the magnitude of the down-regulation. We speculate that ionizing radiation eventually causes sperm damage by damaging spermatogonia.

Ionizing radiation can induce apoptosis of intestinal crypt cells, but the differentiated villi cells are less sensitive to radiation. After the body receives a certain dose of radiation, crypt epithelial cells undergo temporary proliferation inhibition, apoptosis, degeneration, necrosis and other pathological changes, so that the renewal of the villous epithelium become unsustainable, thereby destroying the integrity of the epithelial structure (52). In this study, the small intestinal villus was shortened induced by IR, with apoptosis of a large number of intestinal epithelial cells, while GSPs alleviate these injuries. Ionizing radiation can hinder the differentiation of intestinal stem cells into various functional cells, cause extensive necrosis of intestinal epithelial cells, damage the mechanical and immune barriers of intestinal epithelium, and cause severe gastrointestinal bleeding, diarrhea, bacteremia, and electrolyte disturbances, etc. (2, 3, 8). In this study, when the abdomen was exposed to high-dose ionizing radiation, the feces of the mice decreased, and the feces appeared to be hydrated, indicating that diarrhea occurred after the intestinal barrier was damaged. Some mice died within 1–2 weeks after IR. Most of the mice in the IR+GSPs group survived, suggesting that GSPs have obvious radioprotective effects on intestines.

Reactive oxygen species (ROS) are important in radiation-induced biological damage (53). Proanthocyanidins are currently the most efficient free radical scavenger in plant extracts. Proanthocyanidins can quickly neutralize ROS and reduce radiation damage. In this study, we found that proanthocyanidins significantly reduced ROS levels in TM3 and HIEC cells after radiation. *In vivo*, we found that proanthocyanidins can improve the oxidative stress state of the testes and intestines of mice after ionizing radiation, increasing the content of SOD and GSH, and reducing the content of MDA. Therefore, proanthocyanidins play a significant radioprotective effect in the testes and intestines through antioxidant effects.

After γ-ray or ultraviolet radiation, ROS in the cell will increase, which will activate the MAPK signaling pathway and induce cell apoptosis (54–58). Inhibiting the MAPK signaling pathway can reduce the damage of normal cells after being irradiated by gamma rays or ultraviolet rays (59, 60). Phloridin plays a protective role against ultraviolet-induced skin damage by inhibiting the P38 and JNK signaling pathways (32). Rhein protects against radiation-induced acute enteritis by inhibiting the P38 MAPK signaling pathway (61). In this study, ionizing radiation also activated the MAPK signaling pathway in the testes and intestines, and up-regulated the expression of p-P38 and p-JNK, while GSP can significantly inhibit the expression of p-JNK and p-P38, thereby protecting testis and intestines against damage induced by ionizing radiation. Therefore, proanthocyanidins play a significant role in radioprotection in the testes and intestines by inhibiting the MAPK signaling pathway.

## Conclusion

This study shows that GSPs have radioprotective effects on the testes and intestines, which may be related to the antioxidant effects of GSPs and the inhibition of MAPK signaling pathways. GSPs are expected to be a safe and effective radioprotective drug.

## Data Availability Statement

The raw data supporting the conclusions of this article will be made available by the authors, without undue reservation.

## Ethics Statement

The animal study was reviewed and approved by Laboratory Animal Center of Naval Medical University, Shanghai.

## Author Contributions

HS participated in study design, data collection, statistical analysis, data interpretation, manuscript preparation, and literature search. JH participated in data collection and literature search. CL participated in data collection. FC participated in data interpretation, and funds collection. YH participated in study design, statistical analysis, data interpretation, and funds collection. All authors read and approved the final manuscript.

## Funding

This work was supported in part by the grants from Zhejiang Provincial Natural Science Foundation of China (No. LQ19H220001), National Natural Science Foundation of China (No. 81803168), and Jiaxing Medical Key Discipline (No. 2019-fc-06).

## Conflict of Interest

The authors declare that the research was conducted in the absence of any commercial or financial relationships that could be construed as a potential conflict of interest.

## Publisher's Note

All claims expressed in this article are solely those of the authors and do not necessarily represent those of their affiliated organizations, or those of the publisher, the editors and the reviewers. Any product that may be evaluated in this article, or claim that may be made by its manufacturer, is not guaranteed or endorsed by the publisher.
